# Fatty Acid Composition of *Cannabis sativa*, *Linum usitatissimum* and *Camelina sativa* Seeds Harvested in Lithuania for Food Use

**DOI:** 10.3390/foods10081902

**Published:** 2021-08-16

**Authors:** Violeta Razmaitė, Vidmantas Pileckas, Saulius Bliznikas, Artūras Šiukščius

**Affiliations:** Department of Animal Breeding and Reproduction, Animal Science Institute, Lithuanian University of Health Sciences, R. Žebenkos 12, 82317 Baisogala, Lithuania; Vidmantas.Pileckas@lsmuni.lt (V.P.); Saulius.Bliznikas@lsmuni.lt (S.B.); Arturas.Siukscius@lsmuni.lt (A.Š.)

**Keywords:** fatty acids, seeds, nutrients, oil crops, hemp, flax, camelina

## Abstract

The objective of this study was to determine species-associated differences in the seed proximate and fatty acid composition of three traditional oil crop species, hemp (*Cannabis sativa*), flax (*Linum usitatissimum*), and camelina (*Camelina sativa*), and the sowing season of camelina harvested under Lithuanian farming conditions for food use. Camelina seeds had the highest (*p* < 0.001) content of protein, oil, and sugar contents compared to both dehulled hemp and flax seeds. The amounts of protein and oil in camelina seeds were considerably increased by their summer cultivar, which showed higher (*p* < 0.001) contents of protein and oil than winter cultivars. However, the highest and lowest *(p* < 0.001) fiber content was found in flax seeds and camelina seeds, respectively. Camelina seeds showed considerably higher and lower (*p* < 0.001) proportions of total monounsaturated (MUFA) and polyunsaturated (PUFA) fatty acids, respectively, compared with hemp and flax. The summer cultivar of camelina had higher (*p* < 0.001) proportions of saturated (SFA) and MUFA and lower proportions of PUFA compared with winter cultivars. Hemp seeds had the highest and lowest (*p* < 0.001) proportions of PUFA and MUFA, respectively. The n-6/n-3 PUFA ratio in hemp seeds is optimal (3.79), whereas the use of flax and camelina seeds with their n-6/n-3 ratios of 0.28 and 0.48, respectively, can significantly improve this ratio in the overall diet. The properties of oil crop seeds showed that whole seeds of hemp, flax, and camelina are potentially highly beneficial to human health.

## 1. Introduction

Meat plays a crucial role in human evolution and is an important component of a healthy and balanced diet [1], and meat consumption has long been an indicator of well-being. Despite the fact that today the amount of meat in human diets varies greatly among individuals within societies, meat consumption is rising, and current evidence suggests that increased consumption of meat, especially that of red and processed meats, will adversely affect public health [2]. Therefore, for some nutrient-dense foods, which can increase non-communicable (NCD) disease risk when eaten in excess, dietary recommendations must encourage preventing overconsumption [3,4]. Different epidemiological and clinical studies on the role of dietary fatty acids showed n-3 PUFA exertion of protective effects in coronary heart diseases [5,6,7], cancers, and neurodegenerative disorders [7,8]. As health is becoming an increasingly important value, consumers are seeking foods that offer variety, safety, and especially health benefits. A balanced and healthy diet should be based on available, accessible, affordable, safe, and culturally acceptable food and allow guaranteeing socio-economic and environmental sustainability [9,10]. Therefore, no less important is the consumption of local foods. The importance of foods for health has increased the interest of consumers in different nut, seed, and oil crops, and oils obtained from them are becoming increasingly popular ingredients added to food products [11]. Plant food products from selected ancient crops contain substances with high health benefits. These crops are sources usually rich in fatty acids, sterols, phenolic compounds, and dietary fiber, which have mainly shown the ability to increase satiety and obesity control and prevent other different diseases [12,13,14,15,16,17]. Many oils predominantly supply mostly essential linoleic (LA) and considerably less α-linolenic (ALA) fatty acids; however, greater amounts of ALA are found in flaxseed, hempseed [18,19], and camelina oils [20]. The interest in various seeds and their consumption is steadily growing in Lithuania. The appreciation of global food diversity and acceptance of different foreign edible seeds such as chia, sesame, and others also promote the usage of traditional local edible seeds. Despite the fact that *Camelina sativa* as a food crop was forgotten by the Lithuanian population, paleobotanical and archaeological data indicate that crop cultivation, including *Cannabis sativa* and *Camelina sativa,* appeared in Lithuania in the middle of the Bronze Age [21,22]. The flax (*Linum usitatissimum*) was introduced most recently, during the Late Iron Age [23], and used for fiber and seed production. Notwithstanding that cultivation of *Cannabis sativa* was disallowed, people in Žemaitija (Samogitia), the western region of Lithuania, did not forget an ancient meal of grounded hemp and flax seeds with salt and used it at least on Christmas Eve. Research evidence [24] about anti-inflammatory, anti-hyperalgesic, anti-arthritic, and other clinical effects of cannabidiol (CBD) and resumption of the opportunity to continue the cultivation of industrial hemp highly increased the interest in *Cannabis sativa* and its products. These ancient widely distributed species of oil crops being cultivated in different countries have been studied, and the results regarding their nutritional qualities have been published [14,16,25,26,27,28,29,30]. The obtained differences in the fatty acid composition were explained by the differences between cultivars, as well as differences in genetic diversity, geographical area, soil quality, and climatic conditions [26,31]. Unlike flax and hemp, which are only annual, spring-sowed species, Lithuanian farmers are growing both spring- and autumn-sowed *Camelina cultivars*. Thus, the objective of this study was to determine species-associated differences in the seed proximate and fatty acid composition of three traditional oil crop species (*Cannabis sativa*, *Linum usitatissimum,* and *Camelina sativa*) and the sowing season of camelina harvested under Lithuanian farming conditions for food use.

## 2. Materials and Methods

### 2.1. Chemicals

Sulfuric acid (Sigma-Aldrich Co., St. Louis, MO, USA, 95–97%), hydrochloric acid, sodium hydroxide, potassium chloride, sodium sulfate anhydrous, sodium bicarbonate, potassium sodium tartrate tetrahydrate, copper sulfate pentahydrate, lead (II) acetate trihydrate and lead (II) acetate trihydrate, acetone, and chloroform (Sigma-Aldrich Co., St. Louis, MO, USA, p.a.). Iron (III) sulfate hydrate (Sigma-Aldrich Co., St. Louis, MO, USA, ASC reagent). Potassium permanganate standard solution (Sigma-Aldrich Co., St. Louis, MO, USA, 0.02002 mol/L). Boric acid (AFT, Bratislava, Slovakia, p.a.); Kjeltabs (Velf Scientifica srl, Usmate, Italy). Petroleum ether (Sigma-Aldrich Co., St. Louis, MO, USA, b.p. 40–60 °C). Methanol (Sigma-Aldrich Co., St. Louis, MO, USA, HPLC grade). Sodium methoxide solution (Sigma-Aldrich Co., St. Louis, MO, USA, 25 wt% in methanol). Hexane (Sigma-Aldrich Co., St. Louis, MO, USA, for pesticide residue analysis).

### 2.2. Seeds and Sampling

The study was performed on a total of 62 accessions of flax (*Linum* *usitatissimum*), *Camelina sativa* (spring and winter), and hemp (*Cannabis sativa*) seeds obtained from suppliers approved by the state plant service under the Ministry of Agriculture. The farms were located at a latitude of 54°27′–55°43′ N and at a longitude of 21°12′–23°49′ E. The oilseed flax (*Linum* *usitatissimum*) was represented by a national cultivar Edita. As the local cultivars of hemp and camelina were lost, the seed of *Cannabis sativa* was represented by Finola and *Camelina sativa* by spring Omega and winter Penziak and Luna cultivars introduced in Lithuania.

### 2.3. Proximate Composition of Seeds

The dry matter content was determined [32] by drying samples in an oven at 105 °C until a constant weight was obtained (method no 925.09B; AOAC, 1990). The crude protein content was determined by the Kjeldahl method using the Kjeltec system 1002 apparatus (Foss-Tecator AB, Höganäs, Sweden), and a conversion factor of 6.25 was used to convert total nitrogen to crude protein (method no. 984.13; AOAC, 1990). Crude fat was determined by the Soxhlet extraction method (method no. 920.39C; AOAC, 1990). Ash was determined by incineration in a muffle furnace at 550 °C for 24 h (method no. 920.153; AOAC, 1990). Crude fiber content was determined using the FiberCap 2022 system (Fos-Tecator AB, Höganäs, Sweden) according to method no. 962.09E (AOAC, 1990). The content of sugar was determined by Bertrand’s method [33,34].

### 2.4. Fatty Acid Profiles

The extraction of lipids for fatty acid analysis was performed with a mixture of two volumes of chloroform and one volume of methanol as described by Folch et al. [35]. Methylation of the samples was performed using sodium methoxide: 5 mL of 25 wt% solution in methanol was added to the sample and stirred. After 1 h, 7 mL HCL, 6 mL hexane, and 2 mL H_2_O were added. The top layer was transferred into a new test tube and evaporated. Fatty acid methyl esters were prepared according to the procedure described by Chistopherson and Glass [36]. The FAMEs were analyzed using a gas liquid chromatograph (GC-2010 SHIMADZU, Kyoto, Japan) fitted with a flame ionization detector. The separation of the methyl esters of fatty acids was affected on the capillary column Rt 2560 (100 m × 0.25 mm × 0.2 μm; Restek, Bellefonte, PA, USA) by temperature programming from 160–230 °C. The temperatures of the injector and detector were held at 240–260 °C, respectively. The rate of flow of carrier gas (nitrogen) through the column was 0.79 mL/min. The peaks were identified by comparison with the retention times of the standard fatty acid methyl esters “37 Component FAME Mix” and trans FAME MIX k 110 (Supelco, Bellefonte, PA, USA). The relative proportion of each fatty acid was expressed as the relative percentage of the sum of the total fatty acids using “GC solution” software for Shimadzu gas chromatograph workstations.

### 2.5. Lipid Quality Indices

Lipid quality indices, i.e., atherogenic index (AI) and thrombogenic index (TI), were calculated according to Ulbricht and Southgate [37]. AI = [(4 × C14:0) + C16:0]/[(n-6PUFA + n-3PUFA) + MUFA]; TI = [C14:0 + C16:0 + C18:0]/[0.5 × MUFA + 0.5 × n-6 PUFA + 3 × n-3 PUFA + (n-3/n-6 PUFA)]. The hypocholesterolemic/hypercholesterolemic (h/H) ratio was calculated according to Fernández et al. [38]. h/H = [C18:1n-9 + C18:1n-7 + C18:2n-6 + C18:3n-6 + C18:3n-3 + C20:3n-3 + C20:4n-6 + C22:2n-6]/[C14:0 + C16:0]. The peroxidizability index (PI) was determined according to Du et al. [39]. PI = (monoenoate × 0.025) + (dienoate × 1) + (trienoate × 2) + (tetraenoate × 4).

### 2.6. Statistical Analysis

The data were subjected to the analysis of variance in general linear (GLM) procedure in IBM SPSS Statistics 22 with LSD tests to determine the significance of differences of means between the groups. The GLM models included fixed factors of seed species and the sowing season of camelina cultivars. The differences were regarded as significant when *p* < 0.05.

## 3. Results and Discussion

### 3.1. Proximate Composition

The pro-health effect of oilseed consumption is influenced not only by its high content of oil and essential unsaturated fatty acids but also by other nutrients such as proteins, microelements, antioxidants, and fiber [11,40]. Camelina seeds had the highest (*p* < 0.001) content of protein compared both to dehulled hemp and flax seeds, but all these seeds were rich in protein (Table 1). The highest (*p* < 0.001 and *p* < 0.01) oil and sugar contents were also found in the seeds of camelina compared with the hemp and flax, respectively.

However, in the present study, camelina seeds had lower contents of protein and lipids than the contents reported by other authors [20]. The proximate composition of whole flax seeds corresponded to the ranges described by some authors [40], but our flax seeds had more protein than in the study of Bozan and Temelli [41]. Hemp seeds harvested in Lithuania also had slightly lower contents of crude fat and ash compared with the studies presented by Farinon et al. [16], but the content of crude protein corresponded to the range level reported in the reviewed studies. The differences in the seed composition might have been detected not only because of different cultivars used in the present study and in the studies of other authors but also because of the differences in seed-growing environments. The research of most other authors was conducted in the countries south of Lithuania with different soil quality and climatic conditions, including different air and soil temperatures, precipitation, and harvesting time [26,31,42]. Krzyżaniak et al. [42] have reported the effect of sowing date, beginning and end of flowering, maturity, and harvest dates on the protein and oil contents of camelina in a neighboring country.

The amounts of protein and oil in camelina seeds were considerably increased by their summer cultivar, which showed higher (*p* < 0.001) contents of protein and oil than winter cultivars. Although all plant-derived food has fiber, the total consumption of fiber in current diets is low. Consumption of whole oil crop seeds may have an impact on the intake of fiber. As dietary fiber has been shown to exert an effect on the gut microbiota and its metabolites and thus is hypothesized to lower the risk of coronary heart disease, diabetes, some cancers, and obesity [43,44], all seeds could be a source of fiber intake. However, the highest (*p* < 0.001) fiber content was found in flax seeds and the lowest (*p* < 0.001) in camelina seeds. The highest (*p* < 0.001) ash content, which is an indicator of mineral content, was found in hemp seeds.

### 3.2. Saturated Fatty Acids

The seeds of all oil crop species showed low percentages of saturated fatty acids (Table 2). As the authors have reported variations in the fatty acid composition among different cultivars and lines of oilseed crops [42,45,46], there are differences between the results obtained in the present study and the results reported by other authors. Camelina seeds had a lower percentage of SFA compared with ten different spring genotypes cultivated in Poland [44]. Hemp seeds had a higher SFA proportion than the hemp seeds in the study of Da Porto et al. [47] but a lower proportion than in the other Italian study of Siano et al. [28] and reported by Schultz et al. [45]. Flax seeds exhibited either lower or higher SFA compared to the genotypes of different origins cultivated in Turkey [46]. The most abundant fatty acids were unfavorable palmitic (C16:0) followed by a more favorable for consumers stearic (C18:0) fatty acid, and this is in agreement with other previous reports [25,26,27,28,48]. Regarding saturated fatty acids, some authors [16,25,26] have reported from the saturated fatty acids only the presence of most abundant palmitic and stearic acids or even only the palmitic acid [42]. The review of the studies on human health [49] showed that individual saturated fatty acids have different effects: acids from C12:0 to C18:0 raise LDL-cholesterol and induce insulin resistance, leading to diabetes, promoting inflammation, and increasing the risk of cardiovascular disease (CVD), with C14:0 having the greatest effect and C18:0 having a minor effect. Camelina seeds showed the lowest (*p* < 0.001) percentages of total saturated fatty acids (SFA), including individual C16:0 and C18:0 fatty acids, compared with hemp and flax seeds. Flax had lower (*p* < 0.001) percentages of these acids than hemp. Flax seeds had the lowest (*p* < 0.001) percentages of arachidic (C20:0), behenic (C22:0), and lignoceric (C24:0) acids, while camelina had higher (*p* < 0.001) and lower (*p* < 0.001) percentages of C20:0 and C24:0, respectively, than hemp. Summer camelina had a higher (*p* < 0.001) proportion of SFA, including most individual fatty acids, than winter cultivars.

### 3.3. Monounsaturated Fatty Acids

Oil crop seeds had higher proportions of monounsaturated fatty acids (MUFA) than SFA and the differences in MUFA composition between the oil crop species were larger than in SFA. The European Food Safety Authority (EFSA) has reported that cis-monosaturated acids, which were detected in the present study, have no known specific role in preventing or promoting diet-related diseases [50]. Camelina seeds had relatively, 42.58%, and even 2.8 times higher (*p* < 0.001) total MUFA than flax and hemp, respectively (Table 3). Flax seeds had relatively, 62.9%, higher (*p* < 0.001) proportion of MUFA than hemp seeds. MUFA proportion in camelina seeds was lower than that reported for different genotypes in Poland, including Omega, which was also used in the present study [42]. In some other studies, the proportions of MUFA were shown to be higher [28,45] or quite similar [47] to those in the present study.

The most abundant individual fatty acid in all crops was oleic (C18:1 n-9) acid, and this is consistent with the results reported by other authors [25,27,28,47]. Some of these authors [28] have analyzed only the presence of oleic fatty acid in hemp. In the current study, C18:1n-9 exhibited the highest and lowest (*p* < 0.001) proportions in flax and hemp seeds, respectively. Gadoleic fatty acid (C20:1) was the next abundant acid in camelina, and this is in agreement with the findings of other authors [44]. Hemp and, particularly, flax seeds had very small proportions of this fatty acid. Camelina had also C22:1 and C24:1 fatty acids, whereas in hemp and flax seeds, these fatty acids were not detected. The summer cultivar of camelina had relatively, 2.9%, more (*p* < 0.001) MUFA compared with winter cultivars.

### 3.4. Polyunsaturated Fatty Acids

The highest and lowest (*p* < 0.001) proportions of polyunsaturated fatty acids (PUFA) were detected in hemp and camelina seeds, respectively (Table 4). Hemp seeds grown in Lithuania showed higher proportions of PUFA than in different studies reviewed by Siano et al. [28] and Farinon et al. [16] but lower PUFA compared with the results obtained by Da Porto et al. [47]. Camelina seeds demonstrated higher proportions of PUFA compared with those obtained for different cultivars in Poland [42]. Increased growing temperature can reduce the content of polyunsaturated fatty acids in different oilseed crops [31]. Although oil crop cultivation temperatures were not recorded in the present study and likewise by many other authors, they are likely to be higher in southern countries than in Lithuania. However, this cannot explain the higher proportions of PUFA obtained in Italian hemp [47]. Rodríguez-Rodríguez et al. [26] have detected fatty acid variation of camelina seeds at different developmental stages (days after flowering). In the present study, the most abundant fatty acid in flax and camelina was α-linolenic, ALA (C18:3n-3), followed by linoleic, LA (C18:2n-6), fatty acid, whereas in hemp seeds, conversely, the most abundant fatty acid was C18:2n-6 followed by C18:3n-3. Both these fatty acids are essential. ALA is the substrate for the synthesis of bioactive very-long-chain n-3 PUFAs such as eicosapentaenoic (EPA; C20:5n-3), docosapentaenoic (DPA; C22:5n-3), and docosahexaenoic (DHA; C22:6n-3) fatty acids [51,52].

The proportion of ALA in camelina was 2.3 times, and in flax almost 3.4 times, higher (*p* < 0.001) than in hemp. Hemp appeared to have the highest (*p* < 0.001) proportion of γ-linolenic (C18:3n-6) fatty acid compared with camelina and flax seeds. Grand proportions of ALA in all seeds compared to the proportions of this acid in meats are very impressive; however, overall fatty acid diversity was lower [53]. Only eight, six, and five polyunsaturated fatty acids were found in the lipids of camelina, hemp, and flax seeds, respectively, whereas fifteen polyunsaturated fatty acids were found in different tissues of red deer using the same methods. None of the important very-long-chain fatty acids such as EPA, DPA, and DHA were detected. Arachidonic fatty acid (C20:4n-6, ARA) was not detected in flax seeds. In hemp and camelina seeds, ARA was found only in trace amounts. Arachidonic acid and metabolites play important physiological roles in human health and diseases. However, ARA can be provided to humans not only by the consumption of dietary food that contains high levels of ARA but also through the linoleic acid [54]. Although EFSA [50] proposes not to set a tolerable upper intake level for total or any of the n-6 PUFAs, there is research evidence [52] to suggest that reduction in the dietary intake of LA and ARA (arachidonic C20:4n-6 fatty acid), together with an increase in n-3 long-chain PUFAs, would benefit most consumers. Winter camelina cultivars had higher (*p* < 0.001) proportions of PUFA, including C18:3n-3, eicosatrienoic (C20:3n-3), and C18:3n-6 fatty acids.

### 3.5. Fatty Acid Ratios and Lipid Quality Indices

With the aim to lower serum cholesterol, recommendations for nutrition suggested to reduce the levels of saturated fatty acids and replace them with polyunsaturated fatty acids in the diet [52,55], and the target for the ratio of PUFA to SFA is 0.4 or above [56]. However, other authors reported that a high PUFA/SFA ratio diet enhances oxidative stress because PUFA are highly susceptible to lipid peroxidation and indicated that a PUFA/SFA ratio of 1.0–1.5 and a PI value of 80–90 in the diet are within a favorable range to reduce the risk of CVD. The PUFA/SFA ratios in all seeds were considerably higher, with the highest (*p* < 0.001) value in hemp seeds (Table 5).

Lower PUFA/SFA ratio in hemp was estimated by Siano et al. [28], whereas other authors [47] have reported a higher (10.5) ratio in hemp oil. The addition of all seeds can improve the PUFA/SFA ratio of the overall diet. Recent dietary guidelines are focused on n-6 and n-3 PUFA balance. The recommendations of Bellagio’s report on healthy agriculture, healthy nutrition, and healthy people indicated that the ratio (4:1) of n-6 PUFA to n-3 PUFA in the diet should be the goal [56,57]. It can be observed that the n-6/n-3 PUFA ratio in hemp is the highest (*p* < 0.001) compared with camelina and flax and is fully consistent with dietary guidelines. The considerably lower n-6/n-3 PUFA ratio in camelina and particularly in flax seeds can improve the ratio of the overall diet, which is currently characterized by a n-6/n-3 PUFA ratio of about 10:1 [28]. Due to the low proportion of saturated fatty acids in all seeds, their atherogenic (AI) and thrombogenic (TI) indexes are similar, with slightly higher (*p* < 0.001) values in hemp. However, the best hypocholesterolemic/hypercholesterolemic (h/H) ratio was found also in the hemp seeds. Low lipid quality indices show that the seeds of oil crops are neither non-atherogenic nor thrombogenic and have a very high hypocholesterolemic effect. The highest and lowest (*p* < 0.001) peroxidizability index (PI) was estimated in flax and hemp seeds, respectively. The PI index of all seeds was higher than that indicated by Kang et al. [55] as the most favorable range (80–90) to reduce the risk of CVD. It is well-known that PUFAs and particularly ALA are highly susceptible to oxidation [58], which increases the risk of oil deterioration. Although in the present study, the PI index shows poor oxidative stability of oils, it is known that whole flaxseeds [59] and likely other oil crop seeds are chemically stable. The consumption of whole oil crop seeds can help to avoid oil deterioration and, consequently, detrimental health effects.

Supplementary data regarding gas liquid chromatography chromatograms of the methyl esters from *Camelina sativa*, *Cannabis sativa* and *Linum usitatissimum* seeds can be found in Figures S1–S3, respectively.

## 4. Conclusions

The cultivars of industrial hemp, flaxseed, and camelina grown in Lithuania produce seeds that contain amounts of protein, lipids, and fiber typical for their species. Although camelina showed the largest diversity in monounsaturated and polyunsaturated fatty acid composition, the oil of all seeds is primarily made up of the two essential linoleic and α-linolenic acids observed in different ratios. The PUFA/SFA ratios in all seeds were considerably higher than recommended for a healthy diet, with the highest value in hemp seeds, whereas the lowest n-6/n-3 PUFA ratio was found in flax seeds. Lipid quality indices exhibited that all seeds are neither atherogenic nor thrombogenic but highly hypocholesterolemic. The properties of oil crop seeds showed that the addition of whole hemp, flax, and camelina seeds can improve dietary wellness.

## Figures and Tables

**Table 1:** Seed proximate composition.

Variables	Oil Crop Species			Cultivars of Camelina	
	Camelina	Hemp	Flax	Summer	Winter
Dry matter, %	92.21 ^a,e^ ± 0.14	93.17 ^f^ ± 0.16	92.79 ^b^ ± 0.23	92.45 ^E^ ± 0.06	91.96 ^F^ ± 0.04
Crude protein, %	23.35 ^e^ ± 0.21	22.17 ^f^ ± 0,24	21.51 ^f^ ± 0.35	24.12 ^E^ ± 0.10	22.57 ^F^ ± 0.07
Crude fat, %	34.28 ^e,c^ ± 0.46	30.95 ^f^ ± 0.53	31.87 ^a^ ± 0.75	36.31 ^E^ ± 0.89	32.24 ^F^ ± 0.63
Fiber, %	23.96 ^e^ ± 0.45	26.48 ^a,f^ ± 0.52	28.77 ^f,b^ ± 0.74	20.12 ± 0.83	27.80 ± 0.58
Ash, %	2.98 ^e^ ± 0.07	4.72 ^f^ ± 0.08	3.18 ^e^ ± 0.12	3.08 ^E^ ± 0.04	2.89 ^F^ ± 0.03
Sugars, %	3.76 ^e,c^ ± 0.10	2.33 ^f^ ± 0.12	3.16 ^e,d^ ± 0.17	3.83 ± 0.23	3.73 ± 0.17

The differences between the means of crop species and between camelina cultivars in the rows with different superscripts differ at ^a,b^ *p* < 0.05; ^c,d^ and ^E,F^ *p* < 0.01; ^e,f^ *p* < 0.001.

**Table 2:** Effects of oil crop species and cultivars of different sowing seasonality on saturated fatty acid (% of total FA) composition in seed lipids.

Fatty Acids	Oil Crop Species			Cultivars of Camelina	
	Camelina	Hemp	Flax	Summer	Winter
C14:0	0.05 ^e^ ± 0.01	0.03 ^f,a^ ± 0.001	0.04 ^f,b^ ± 0.001	0.05 ^E^ ± 0.001	0.48 ^F^ ± 0.001
C16:0	4.94 ^e^ ± 0.03	5.93 ^f,e’^ ± 0.03	5.26 ^f,f’^ ± 0.05	5.1 ^E^ ± 0.03	4.86 ^F^ ± 0.02
C17:0	0.03 ^c,e^ ± 0.002	0.04 ^d^ ± 0.002	0.04 ^f^ ± 0.03	0.03 ± 0.004	0.03 ± 0.003
C18:0	2.10 ^e^ ± 0.03	2.41 ^f,e’^ ± 0.03	4.03 ^f,f’^ ± 0.04	2.20 ^E^ ± 0.02	2.04 ^F^ ± 0.12
C20:0	1.01 ^e^ ± 0.01	0.68 ^f,e’^ ± 0.01	0.12 ^f,f’^ ± 0.01	1.02 ± 0.02	1.00 ± 0.01
C22:0	0.19 ^f^ ± 0.003	0.25 ^f,e’^ ± 0.03	0.09 ^f.f’^ ± 0.01	0.20 ^C^ ± 0.01	0.18 ^D^ ± 0.004
C24:0	0.10 ^e^ ± 0.004	0.10 ^e^ ± 0.004	0.06 ^f^ ± 0.01	0.12 ^C^ ± 0.01	0.10 ^D^ ± 0.004
SFA	8.41 ^e^ ± 0.050	9.45 ^f^ ± 0.06	9.64 ^f^ ± 0.09	8.72 ^E^ ± 0.04	8.25 ^F^ ± 0.03

The differences between the means of crop species and between camelina cultivars in the rows with different superscripts differ at ^a,b^ *p* < 0.05; ^c,d^ and ^C,D^ *p* < 0.01; ^e,f;e’,f’^ and ^E,F^ *p* < 0.001. SFA = sum of all identified saturated fatty acids.

**Table 3:** Effects of oil crop species and camelina cultivars of different sowing seasonality on monounsaturated fatty acid (% of total FA) composition in seed lipids.

Fatty Acids	Oil Crop Species			Cultivars of Camelina	
	Camelina	Hemp	Flax	Summer	Winter
C16:1n-9	0.03 ^e^ ± 0.002	0.02 ^f^ ± 0.002	0.02 ^f^ ± 0.003	0.04 ^E^ ± 0.003	0.02 ^F^ ± 0.002
C16:1n-7	0.08 ^e^ ± 0.001	0.10 ^f,e’^ ± 0.001	0.06 ^f,f’^ ± 0.002	0.08 ± 0.002	0.08 ± 0.001
C17:1n-9	0.02 ^a^ ± 0.001	0.018 ^e^ ± 0.001	0.023 ^b,f^ ± 0.01	0.02 ± 0.001	0.02 ± 0.001
C18:1n-9	16.03 ^e^ ± 0.11	10.07 ^f,e’^ ± 0.13	17.37 ^f,f’^ ± 0.19	16.66 ^E^ ± 0.11	15.71 ^F^ ± 0.08
C18:1n-7	0.50 ^a,c^ ± 0.05	0.73 ^d^ ± 0.06	0.74 ^b^ ± 0.08	0.42 ± 0.11	0.54 ± 0.08
C20:1n-11	12.64 ^e^ ± 0.06	0.30 ^f^ ± 0.08	0.09 ^f^ ± 0.11	12.65 ± 0.16	12.64 ± 0.11
C22:1n-9	1.86 ^e^ ± 0.02	0.00 ^f^ ± 0.02	0.00 ^f^ ± 0.03	1.90 ± 0.04	1.84 ± 0.03
C24:1	0.69 ^e^ ± 0.01	0.00 ^f^ ± 0.01	0.00 ^f^ ± 0.015	0.69 ± 0.02	0.70 ± 0.02
MUFA	31.85 ^e^ ± 0.13	11.23 ^f,e^ ± 0.16	18.29 ^f,f’^ ± 0.22	32.46 ^E^ ± 0.21	31.54 ^F^ ± 0.15

The differences between the means of crop species and between camelina cultivars in the rows with different superscripts differ at ^a,b^ *p* < 0.05; ^c,d^ *p* < 0.01; ^e,f;e’,f’^ and ^E,F^ *p* < 0.001. MUFA = sum of all identified monounsaturated fatty acids.

**Table 4:** Effects of oil corn species and camelina cultivars of different sowing seasonality on polyunsaturated fatty acid (% of total FA) composition in seed lipids.

Fatty Acids	Oil Crop Species			Cultivars of Camelina	
	Camelina	Hemp	Flax	Summer	Winter
C9,t12-C18:2	0.03 ^e^ ± 0.002	0.09 ^f,e’^ ± 0.003	0.44 ^f,f’^ ± 0.004	0.03 ± 0.002	0.03 ± 0.002
C18:2n-6	17.49 ^e^ ± 0.07	58.99 ^f,e’^ ± 0.08	15.46 ^f,f’^ ± 0.12	17.45 ± 0.06	17.52 ± 0.04
C18:3n-6	0.13 ^e^ ± 0.07	2.70 ^f,e’^ ± 0.08	0.18 ^f’^ ± 0.12	0.12 ^A^ ± 0.002	0.13 ^B^ ± 0.002
C18:3n-3	38.94 ^e^ ± 0.21	16.68 ^f,e’^ ± 0.26	56.22 ^f,f’^ ± 0.36	38.21 ^E^ ± 0.19	39.3 ^F^ ± 0.14
C20:2n-6	1.54 ^e^ ± 0.02	0.84 ^f,e’^ ± 0.02	0.00 ^f,f’^ ± 0.03	1.41 ^E^ ± 0.01	1.60 ^F^ ± 0.01
C20:3n-3	1.40 ^e^ ± 0.02	0.00 ^f,e’^ ± 0.19	0.17 ^f,f’^ ± 0.03	1.37 ± 0.04	1.41 ± 0.03
C20:4n-6	0.09 ^a^ ± 0.02	0.02 ^b^ ± 0.02	0.00 ^b^ ± 0.03	0.10 ± 0.05	0.08 ± 0.03
C22:2n-6	0.14 ^e^ ± 0.01	0.00 ^f^ ± 0.01	0.00 ^f^ ± 0.01	0.12 ± 0.02	0.15 ± 0.01
PUFA	59.74 ^e^ ± 0.17	79.32 ^f,e’^ ± 0.21	72.07 ^f,f’^ ± 0.29	58.82 ^E^ ± 0.22	60.21 ^F^ ± 0.15

The differences between the means of crop species and between camelina cultivars in the rows with different superscripts differ at ^a,b^ and ^A,B^ *p* < 0.05; ^e,f;e’,f’^ and ^E,F^ *p* < 0.001; PUFA = sum of all identified polyunsaturated fatty acids.

**Table 5:** Fatty acid ratios and lipid quality indices in seed oil from different oil crop species and camelina cultivars of different sowing seasonality.

Variables	Oil Crop Species			Cultivars of Camelina	
	Camelina	Hemp	Flax	Summer	Winter
PUFA/SFA	7.11 ^e,c^ ± 0.06	8.40 ^f^ ± 0.07	7.50 ^d,e^ ± 0.10	6.75 ^E^ ± 0.05	7.30 ^F^ ± 0.04
n-6/n-3	0.48 ^e,a^ ± 0.04	3.79 ^f^ ± 0.05	0.28 ^e,b^ ± 0.07	0.49 ± 0.003	0.48 ± 0.002
AI	0.06 ^e^ ± 0.00	0.07 ^f^ ± 0.00	0.06 ^e^ ± 0.001	0.06 ^E^ ± 0.00	0.055 ^F^ ± 0.00
TI	0.05 ^e^ ± 0.001	0.10 ^f^ ± 0.001	0.05 ^e^ ± 0.001	0.05 ^E^ ± 0.00	0.046 ^F^ ± 0.00
h/H	14.97 ^e^ ± 0.08	14.97 ^e^ ± 0.1	17.05 ^f^ ± 0.14	14.45 ^E^ ± 0.08	15.24 ^F^ ± 0.05
PI	101.26 ^e^ ± 0.34	99.05 ^f,e’^ ± 0.42	129.09 ^f,f’^ ± 0.59	99.64 ^E^ ± 0.48	102.07 ^F^ ± 0.34

The differences between the means of crop species and between camelina cultivars in the rows with different superscripts differ at ^a,b^ *p* < 0.05; ^c,d^ *p* < 0.01; ^e,f;e’,f’^ and ^E,F^ *p* < 0.001. PUFA/SFA = ratio of PUFA to SFA, n-6/n-3 = ratio of n-6 PUFA to n-3 PUFA, AI = atherogenic index, TI = thrombogenic index, h/H = hypocholesterolemic/hypercholesterolemic ratio, PI = peroxidizability index.

## Data Availability

Data are contained within this article and the Supplementary Material.

## Supplementary Materials

The following are available online at https://www.mdpi.com/article/10.3390/foods10081902/s1, Figure S1: Gas liquid chromatograpgy chromatogram 277 of the metil esters from *Camelina sativa* seeds; Figure S2: Gas liquid chromatograpgy chromatogram 257 of the metil esters from *Cannabis sativa* seeds; Figure S3: Gas liquid chromatograpgy chromatogram 274 of the metil esters from *Linum usitatissimu*m seeds.
