# Supplementary material for: Fatty Acid Composition of Cannabis sativa, Linum usitatissimum and Camelina sativa Seeds Harvested in Lithuania for Food Use
Source: Foods. 2021 Aug 16;10(8):1902. doi: 10.3390/foods10081902 (PMC8394818; doi:10.3390/foods10081902)
Supplement: Supplementary file 1 [file foods-10-01902-s001.zip › foods-1296089-supplementary.pdf]

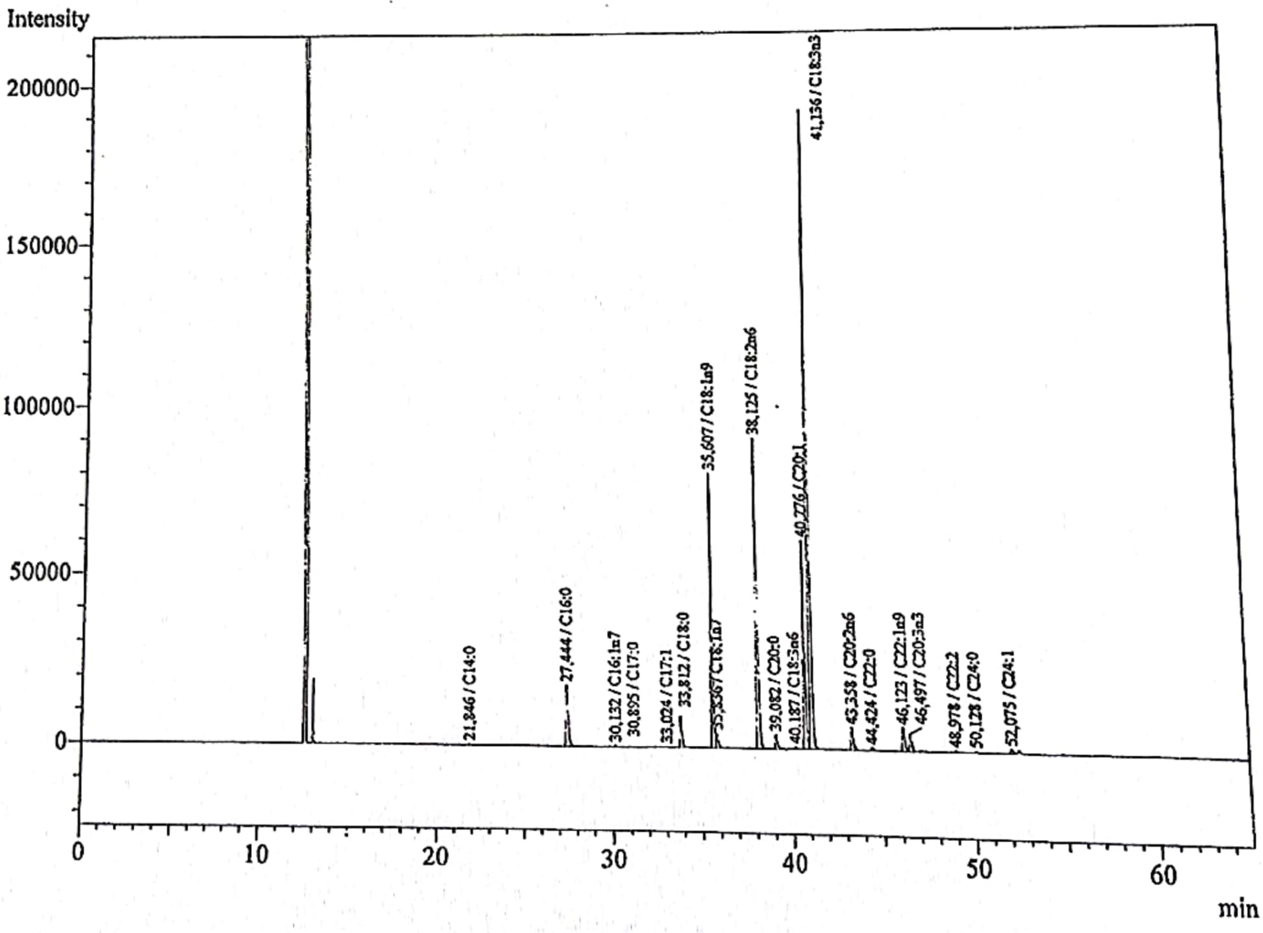

Figure S 1: Gas liquid chromatograpgy chromatogram 277 of the metil esters from Camelina sativa seeds.

Scanned with CamScanner

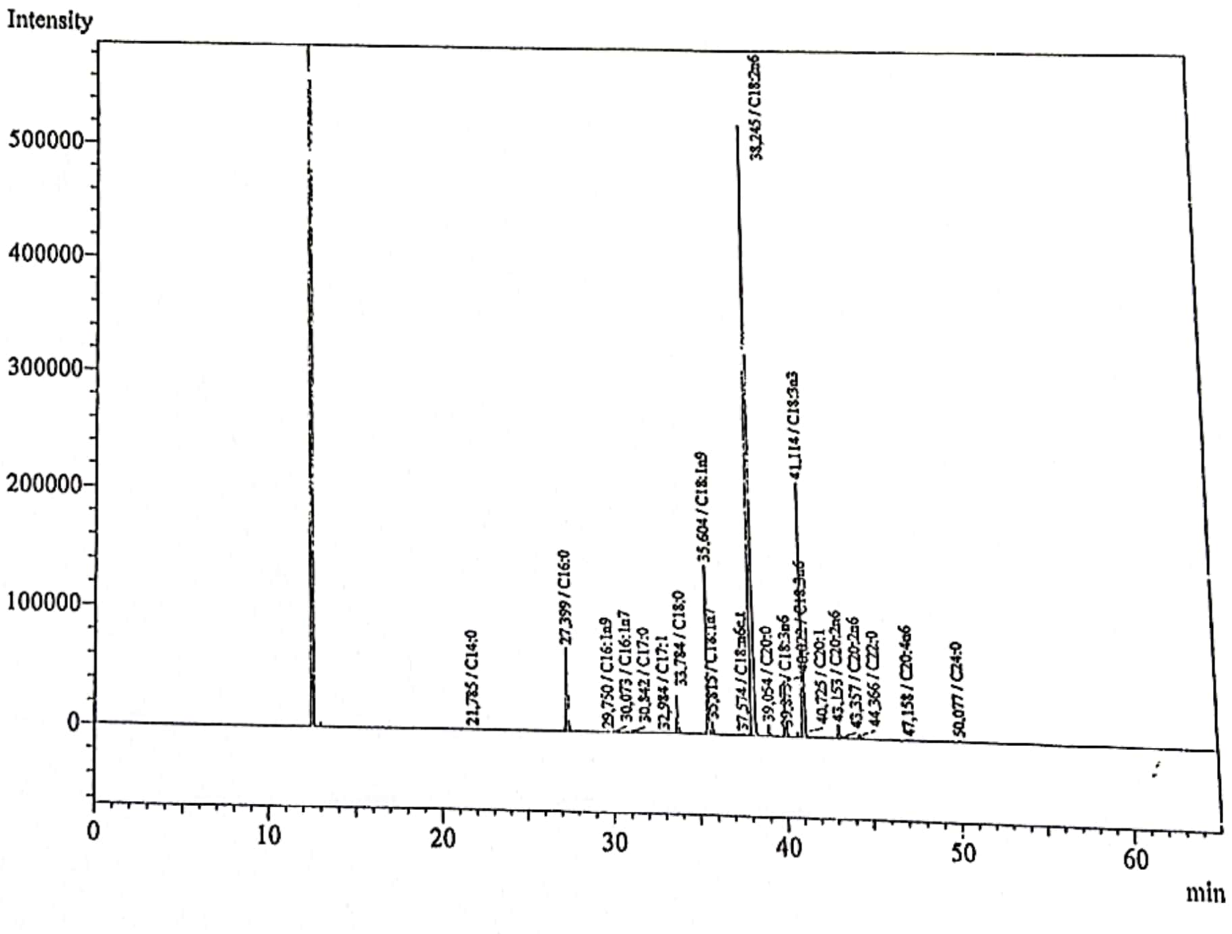

Figure S 2: Gas liquid chromatograpgy chromatogram 257 of the metil esters from Cannabis sativa seeds.

Scanned with CamScanner

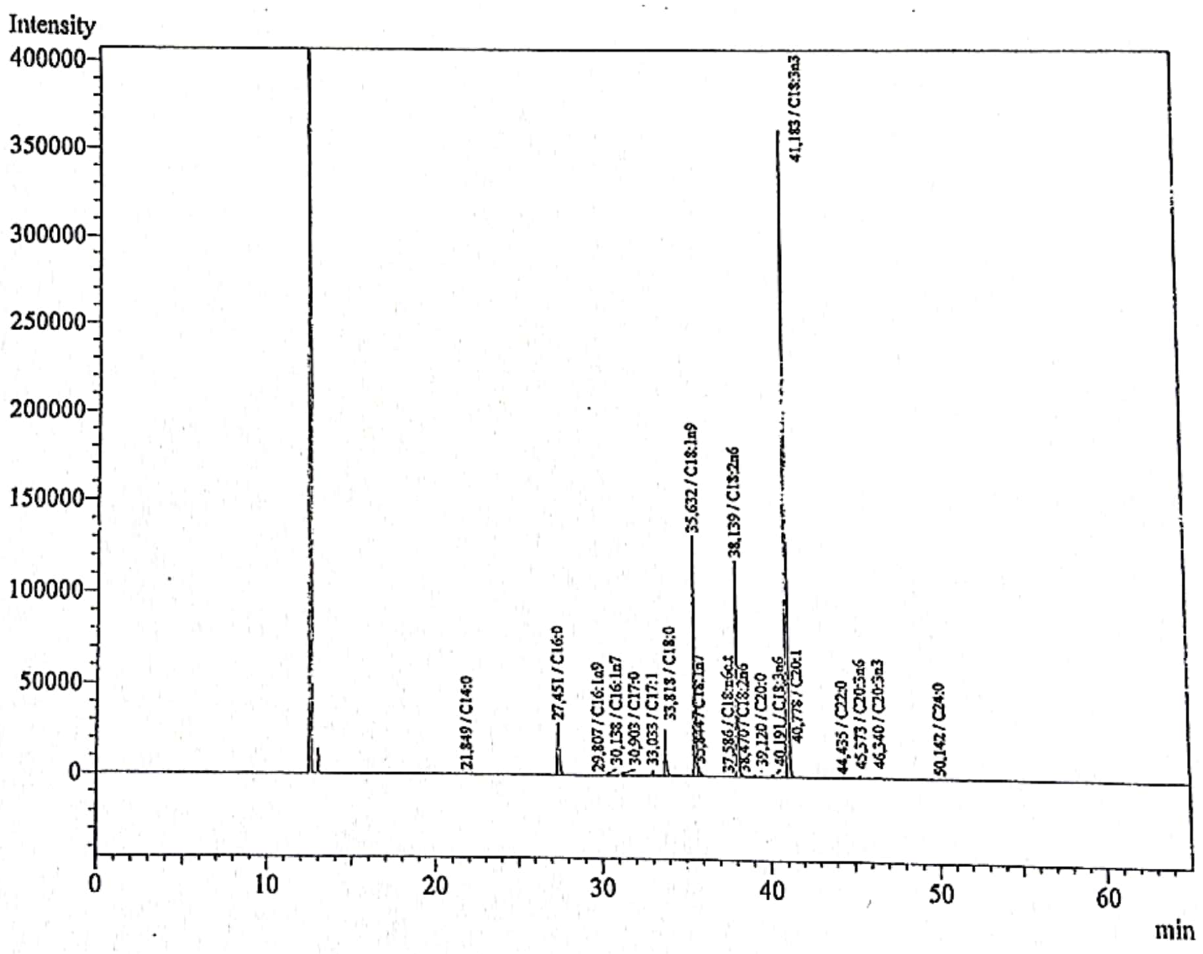

Figure S 3: Gas liquid chromatograpgy chromatogram 274 of the metil esters from Linum usitatissimum seeds.

Scanned with CamScanner
